# Serum vs. tissue cytokine dysregulation in SLE-associated myocarditis: a hypothesis from an African cohort perspective

**DOI:** 10.1186/s10020-026-01504-6

**Published:** 2026-05-21

**Authors:** Tracey Ollewagen, Riette du Toit, Sumanth Karamchand, Anton F Doubell, Philip G Herbst, Carine Smith

**Affiliations:** 1 Experimental Medicine Research Group, Department of Medicine, Stellenbosch University, Cape Town, South Africa; 2 Division of Rheumatology, Department of Medicine, Stellenbosch University, Cape Town, South Africa; 3 Division of Cardiology, Department of Medicine, Stellenbosch University, Cape Town, South Africa

**Keywords:** Interleukin-6, Cytokines, Lupus myocarditis, Fibrosis, Dysregulation, Autoimmunity, Comorbidity, Systemic lupus erythematosus

## Abstract

**Background:**

Clinical lupus myocarditis (LM) occurs in 5–10% of systemic lupus erythematosus (SLE) patients while sub-clinical presenting in approximately 40%. To date, an in-depth cytokine profile of cardiac tissue is lacking, yet vital to understanding disease mechanisms. This study aimed to correlate cardiac tissue inflammatory profile to standard of care (SOC) parameters (serum biomarkers and cardiac MRI (CMR)) in an African mixed-ancestry LM population.

**Methods:**

One control (*n* = 6) and two SLE cohorts, SLE (*n* = 13) and SLE with myocarditis (SLE-M; *n* = 14) were recruited. Serum cytokines were assessed using immunoassays. SLE patients underwent SOC clinical assessments (serology, CMR) and endomyocardial biopsy (EMB, LM only). EMB tissue was immunostained for inflammation, fibrosis and tissue damage.

**Results:**

Systemic inflammation in LM was confirmed with increased serum (s) sTNF-α (*p* < 0.05), sIL-18 (*p* < 0.05), sIL-6 (*p* < 0.01), sIL-10 (*p* < 0.01) and sVCAM-1 (*p* < 0.001). Higher sIL-10 (associated with disease activity) and sVCAM-1 (endothelial activation) were observed in SLE-M vs. SLE (*p* < 0.01). Matching serum and tissue parameters showed no correlation, suggesting a disconnect between circulation and tissue compartments. Functionally contradictory negative correlations between sTNF-α, sVCAM-1 and tissue (t) tIL-6 (both *p* < 0.05) implied a relative lower presence of tIL-6, which would limit conversion from a pro-inflammatory to an anti-inflammatory, reparative state (supported by accompanying low tIL-10). This limited tIL-6 presence was further implicated by negative correlations with CMR inflammation and necrosis/fibrosis (T1, T2). Anti-Ro/La correlated to TGF-β (*p* < 0.05), key in fibrosis development. Fibrosis markers did not correlate with CMR fibrosis suggesting a temporal lag before detectable CMR fibrosis.

**Conclusions:**

Based on presented exploratory data, we hypothesize that inadequate activation of myocardial IL-6 and IL-10 may impair inflammatory regulation. Data suggest that myocardial pro-fibrotic signaling may be detectable at a time point preceding CMR-detectable fibrosis. Purpose-designed research to test this hypothesis of limited IL-6 presence is required to inform on future therapeutic strategies.

**Supplementary Information:**

The online version contains supplementary material available at 10.1186/s10020-026-01504-6.

## Background

Systemic lupus erythematosus (SLE) is a systemic autoimmune disease characterized by multi-organ damage as a result of dysregulated inflammation and immunity. While considered a rare to moderately rare disease, SLE affects an estimated 3.4 million people globally (Siegel and Sammaritano 2024). The chronic nature of the disease, associated morbidity and mortality and need for long-term care places a significant burden on healthcare systems, especially in populations with higher disease prevalence. Patients of African descent tend to develop SLE at a younger age, have higher disease activity, and develop more organ damage than white patients (Essouma and Noubiap 2024).

In SLE, myocarditis may present subclinically (up to 40% of patients), with myocardial involvement evident only on post-mortem studies or through more advanced imaging such as cardiac magnetic resonance (CMR). Evidence of subclinical myocarditis has been reported irrespective of SLE disease activity, clinical or biochemical features of myocardial disease (Bidani et al. 1980; Guo et al. 2018; Panchal et al. 2006). Clinically evident lupus myocarditis (LM) occurs in up to 10% of patients with SLE, typically in the context of recent diagnosis and high SLE disease activity (Dhooria et al. 2020; Tanwani et al. 2018). We have previously reported a similar LM prevalence - and higher mortality (18%) - than other populations, in a South African mixed ancestry SLE patient cohort (Apte et al. 2008; Du Toit et al. 2017).

Current diagnosis of LM entails a combined decision-making process, with consideration of clinical parameters, biomarkers in blood, as well as imaging evidence of myocardial involvement (du Toit et al. 2023). Echocardiography and cardiac magnetic resonance imaging (CMR) is frequently utilized as non-invasive diagnostic tools, providing a valuable assessment of myocardial function and tissue characteristics (Drazner et al. 2025). Endomyocardial biopsy (EMB), a low-risk procedure in experienced hands, is still regarded as the diagnostic gold standard for various causes of myocarditis (Seferović et al. 2021). Yet, few studies have specifically evaluated the role of EMB in LM. An in-depth profile and understanding of the mechanistic interplay and dysregulation within the tissue that underpin myocarditis in SLE remains lacking (Thomas et al. 2017; du Toit et al. 2023).

The underlying pathophysiology of LM is not fully understood, although largely predicted to be immune-complex mediated injury rather than resulting from direct myofibril injury (Wijetunga and Rockson 2002). While studies have assessed cardiac structural changes, immune cell infiltrate and presence of immune complexes (Doria et al. 2005; Kikuchi et al. 2021), analysis of the inflammatory cytokine profile in the cardiac tissue has not yet been performed. In experimental models of human autoimmune diseases, a consensus has been reached in which pro-inflammatory cytokines play a significant role in the initiation and propagation of autoimmune inflammation, while anti-inflammatory cytokines fail in their role of facilitating the resolution of inflammation (Moudgil and Choubey 2011). Additionally, immune complexes are known to trigger the release of pro-inflammatory cytokines at organ level in SLE (Dong et al. 2025). Increased levels of interleukin (IL)-1Ra, IL-17, IL-18 and vascular cell adhesion molecule-1 (VCAM-1) have been reported in the circulation of LM patients (Du Toit et al. 2021) but the cytokine microenvironment at tissue level remains insufficiently characterised.

Despite the fact that African-American ancestry is associated with an increased risk of developing LM and the established negative impact of LM on overall survival and damage accrual in SLE (Apte et al. 2008), almost no data has been generated in the African population (Tiffin et al. 2014; Du Toit et al. 2017). Therefore, to contribute to our understanding of dysregulation at myocardial tissue level and how these relate to clinical observations, we assessed inflammation and fibrosis signaling in a South African mixed ancestry cohort with SLE, in parallel with serum biomarkers and imaging parameters. This involved the extensive tissue assessment of key pro- and anti-inflammatory cytokines involved in general inflammatory regulation, endothelial markers due to microcirculatory disturbances noted in SLE(Vyas et al. 2025) and standard fibrosis markers.

## Methods

### Study design

Ethical approval was obtained from Stellenbosch University Health Research Ethics Committee (reference S18/01/015 and HREC2-2020-13147). Written informed consent was obtained from all participants and all research was conducted in accordance with guidelines of the WHO and the Declaration of Helsinki. Study participants were divided into three groups: two SLE cohorts and a healthy control group. Two SLE cohorts were recruited from Tygerberg Academic Hospital in South Africa - one group (*n* = 14) with clinically evident LM (referred to as SLE-M), and the other (*n* = 13) SLE patients not presenting with clinical or imaging features of LM (referred to as SLE). Inclusion criteria were participants over 18 years of age, fulfilling the 2019 European League Against Rheumatism (EULAR)/American College of Rheumatology (ACR) classification criteria for SLE. A diagnosis of LM included clinical features of myocardial dysfunction attributed to SLE, supported by biochemical evidence of myocardial injury, 2D-echocardiography and CMR as assessed by a rheumatologist and cardiologist experienced in cardiac MRI. Exclusion criteria included patients under 18 years of age, presence of myocardial dysfunction or injury attributed to non-SLE causes including infections, toxins, other systemic disorders, epicardial coronary artery disease or patients with contraindications to CMR. Patients underwent standard of care (SOC) clinical assessment including SLE disease activity (SLEDAI-2 K), laboratory assessments (hematological, biochemical and serological testing), 12-lead electrocardiogram, 2D-echocardiography, CMR, and EMB (EMB only in SLE-M group). A third control cohort of 6 healthy controls was recruited for serum analysis only. The exclusion criteria for healthy controls included individuals with acute/chronic infections, chronic disease or obesity, and use of anti-inflammatory medications.

The imaging data from both SLE patient groups is reported on in detail and from a clinical perspective elsewhere (Karamchund et al., under review). In this paper, our inclusion of the standard of care and imaging data is limited to our analysis of their relationships with our newly added laboratory data informing on tissue level inflammation and fibrosis signaling profiles.

### Standard of care and diagnostic assessments

#### Cardiac magnetic resonance (CMR)

CMR imaging was performed using a Siemens Magnetom Aera 1.5 Tesla scanner (Siemens Healthcare, Erlangen, Germany). All images were acquired in accordance with the 2013 consensus guidelines published by the Society of Cardiovascular Magnetic Resonance (Kramer et al. 2013). Cine images were acquired in the standard cardiac planes (2-chamber, 3-chamber, 4-chamber, and short-axis views) using an electrocardiogram-gated, breath-held balanced steady-state free precession sequence. Non-contrast tissue characterization modules included short tau inversion recovery (STIR) imaging and T1/T2 mapping using a modified-Look Locker inversion recovery sequence. STIR is used to primarily visualize myocardial oedema and inflammation (H-Ici et al. 2012); quantification of T1 allows for the assessment of the degree of myocardial fibrosis not detected by late gadolinium enhancement (LGE), as well as oedema; T2 detects and quantifies myocardial oedema associated with infarction or myocarditis (Tseng et al. 2016). Late gadolinium enhancement images were acquired 10–12 min after contrast injection with 0.2 mmol/L Gadovist (Bayer Inc., Canada). CMR images were post-processed using CVI42 software (version 5.14.3, Circle Cardiovascular Imaging, Canada). This allows for the identification of the location and extent of scarring (Tseng et al. 2016). A morphological, volumetric, and functional analysis was performed using the cine images. Endo- and epicardial borders were drawn on a 3-slice short axis stack for the STIR signal intensity ratio, T1 and T2 relaxation times. Normal T1 and T2 reference ranges using healthy volunteers were previously established and published by our group (Robbertse et al. 2022).

#### Biomarker assessments

All SLE and SLE-M patients underwent standard laboratory investigations including full blood count with differential white cell count, serum urea and creatinine, estimated glomerular filtration rate (eGFR), urine protein creatinine ratio (U-PCR), electrolytes, random serum cholesterol, erythrocyte sedimentation rate (ESR), complement, creatine kinase (CK) and troponin-T. Blood was also screened for auto-antibodies including anti-dsDNA, anti-Ro/SSA and anti-La/SSB antibodies. All biomarker assessments were performed by the National Health Laboratory Services in South Africa according to international standards.

#### Coronary angiography and endomyocardial biopsy (SLE-M patients only)

Coronary angiography of the left and right coronary arteries was performed via radial or femoral artery access as per recommended guidelines at Tygerberg Hospital, as was EMB. Standard right ventricular biopsies (six to eight specimens) were performed via femoral venous access using a 7 French vascular access sheath, a standard introducing sheath, and biopsy forceps (Hassan et al. 2022).

### Analysis of molecular signaling profiles

#### Serum cytokine analysis

Blood was collected in serum separating tubes (SST), allowed to clot for at least 30 min, and centrifuged at 1000 x g for 10 min at room temperature. Serum was removed, aliquoted and frozen at -80 °C for further analysis. Serum TNF-α, IL-1β, IL-1Ra, IL-17a, IL-18, IL-6 and IL-10 were assessed using a Merck Milliplex human cytokine/chemokine/growth factor kit (HCYTA-60 K-07) according to manufacturer’s instructions using a Luminex LX-200 system. Serum VCAM-1 was measured using a solid phase sandwich ELISA kit (DVC00, R&D systems) according to manufacturer’s instructions using a Victor Nivo Multimode Microplate Reader (Perkin Elmer, USA). Data was plotted against a standard curve to determine accurate concentrations. IL-1β and IL-17a concentrations were undetectable in a large number of patients and were therefore excluded from statistical analyses. The same outcome was previously noted in a comprehensive serum cytokine analysis from a LM cohort at the same hospital(Du Toit et al. 2021).

#### Tissue analysis

EMB tissue was placed in optimal cutting temperature (OCT) medium (UltraFreeze, UF1000, Cancer Diagnostics, Inc, USA) and frozen in liquid nitrogen-cooled isopentane (M32631, Sigma-Aldrich, USA) and stored at -80 °C. Tissue was sectioned into 7 μm sections using a Cryostat Microtome (Dakewe 6250, Dakewe Medical, Shenzhen, China) at -20 °C and placed on a poly-L-lysine (P4707, Sigma-Aldrich, USA) pre-coated microscope slide. To facilitate visualisation of general structure of the tissue, sections were stained using a haematoxylin (to identify nuclei) and eosin (to visualize tissue) staining protocol. Briefly, sections were submerged in each reagent for one minute per change in the following order: tap water, Mayer’s hematoxylin (2 changes) (51275, Sigma-Aldrich, USA), warm tap water, phosphate buffered saline (PBS; P4417, Sigma-Aldrich, USA), tap water, eosin (E4009, Sigma-Aldrich, USA), 95% ethanol (3 changes), 100% ethanol (2 changes), and xylene (3 changes) (534056, Sigma-Aldrich, USA). Once stained, cover slips were mounted with DPX mounting media (06522, Sigma-Aldrich, USA).

After acclimating at room temperature for 20 min, sections were fixed in 4% paraformaldehyde (158127, Sigma-Aldrich, USA) for 10 min, washed with PBS with 0.1% Tween-20 (PBS-T)(P1379, Sigma-Alrich, USA) and blocked for 90 min in PBS with 5% donkey serum (S217G, Celtic Diagnostics, South Africa), 20% fetal bovine serum (A5256701, Gibco, Thermo-Fisher Scientific, USA) and 0.2% triton-X-100 (X100, Sigma-Aldrich, USA).

Thereafter, sections were incubated with primary antibodies made up in 5x diluted blocking buffer. Primary antibodies included Vimentin (1:1000, ab8978, mouse monoclonal, clone: RV202, isotype: IgG1, Abcam, UK), VCAM-1 (1:200, MA5-31965, rabbit polyclonal, Invitrogen, USA), TNF-α (1:200, NB600-587, rabbit polyclonal, Novus Biologicals, USA), TGF-β (1:500, ab215715, rabbit monoclonal, clone: EPR21143, isotype: IgG, Abcam, UK), MCP-1 (1:250, NBP1-07035, rabbit polyclonal, Novus Biologicals, USA), IL-1β (1:500, NB600-633, rabbit polyclonal, Novus Biologicals, USA), IL-6 (1:500, NB600-1131, rabbit polyclonal, Novus Biologicals, USA), IL-18 (1:200, PA5-79479, rabbit polyclonal, Invitrogen, USA), IL-10 (1:500, ab34843, rabbit polyclonal, Abcam, UK), IL-1Ra (1:500, PA5-21776, rabbit polyclonal, Invitrogen, USA), BMP-7 (1:400, NBP1-69126, rabbit polyclonal, Novus Biologicals, USA), Claudin-5 (1:200, NBP2-66783, rabbit monoclonal, clone: JM11-22, isotype: IgG, Novus Biologicals, USA), Collagen I (1:100, MA1-26771, mouse monoclonal, clone: COL-1, isotype: IgG1, Invitrogen, USA), Collagen III (1:200, PA1-28870, rabbit polyclonal, Invitrogen, USA), Actin (1:250; NB100-74340, mouse monoclonal, clone: mAbGEa, isotype: IgM kappa, Novus Biologicals, USA). After washing with PBS-T, secondary antibody (AlexaFluor 594 (1:250, A21207, Life Technologies, USA) or AlexaFluor 488 (1:250, A21202, Invitrogen, USA) made up in 5x diluted blocking buffer was added to the sections and incubated for 90 min. After washing with PBS, coverslips were mounted with fluorescent mounting media (S3023, DAKO, Denmark). Images were acquired using a Nikon ECLIPSE Ti2 inverted microscope with NIS Elements D v5.30.02 software. Images were taken using a 40x objective and two fields of view were imaged per section. Immunofluorescent intensities were quantified using integrated density on ImageJ 1.49v software. Prior to sample staining, antibody optimization was performed to determine optimal dilutions. Here, appropriate primary and secondary antibody controls, and negative tissue controls were imaged to determine microscope exposure times suited for imaging of each antibody, as well as autofluorescence (Supp Fig. 1). These settings were carried forward to sample imaging to ensure all signal observed was the selected protein. Only previously validated and commercially available antibodies were used.

### Statistical analysis

Statistical analyses were performed using GraphPad Prism v8.0.2 (http://www.graphpad.com, USA). The ROUT method was used to remove outliers (Q = 1%). All data were analysed for normality with the Shapiro-Wilk test. Due to the majority of data presenting as non-parametric, all data were analysed using non-parametric tools. Column analysis was performed with the Kruskal-Wallis test and Dunn’s multiple comparisons test. Dunn’s test results are presented as p-values after correction for false discovery rate, to limit risk of type I errors in data interpretation. Correlations between parameters were determined by calculation of Spearman’s coefficients. LGE was analysed using Mann-Whitney test. Data are presented as medians ± IQR (interquartile range).

## Results

Thirteen SLE patients (with no symptoms of myocarditis; referred to as SLE) were recruited with a mean age of 32.31 ± 9.65 years. All SLE patients were female, 9 patients were of mixed race and 4 were black. Fourteen SLE-M (SLE with myocarditis) patients were recruited, with a mean age of 30.36 ± 9.15 years. Again, all SLE-M patients were female, 10 of mixed race and 4 black. Healthy controls (*n* = 6) were slightly older (50.2 ± 8.5 years) and included 3 males and 3 females, of which 2 were mixed race and 4 white. Additional profile parameters are presented in Table 1.

**Table 1:** Baseline characteristics of systemic lupus erythematosus (SLE) and lupus myocarditis (SLE-M) patients. Additional SLEDAI-2 K organ manifestations listed in Supp Table 1. Data analysed using Mann-Whitney test

	*SLE*	*SLE-M*	*p-value*
	*n* = 13	*n* = 14	
Age (years); mean ± SD	32.31 ± 9.65	30.36 ± 9.15	
Duration of SLE (weeks); median(IQR)	238(10–652)	6.5(0-215.8)	*p* = 0.096
SLEDAI-2 K scores; median (IQR)	8(3–15)	12.5(8.75–14.25)	*p* = 0.164
aDsDNA (IU/mL); median (IQR)	53(31-191.5)	139(41.5–201)	*p* = 0.284
aRo positive (> 20 IU/ml); n (%)	3/9 (33.3)	6/12 (50)	
aRo titre (IU/ml); median (IQR)	10 (3-71.5)	21 (10–119)	*p* = 0.253
aLa positive (> 20 IU/ml) ; n (%)	2/9 (22.2)	3/12 (25)	
aLa titre (IU/ml); median (IQR)	4 (2.5–15)	6 (2–85)	*p* = 0.441
ANA positive (> 1:80); n (%)	10/13 (77)	14/14 (100)	
ANA titre; median (IQR)	320 (40–960)	960 (320–1280)	*p* = 0.080
aSm positive (> 20 IU/ml); n (%)	6/13 (46)	9/14 (64)	
aSm titre; median (IQR)	30 (6.25–198)	71 (18.5–141)	*p* = 0.241
aRNP positive (> 20 IU/ml); n (%)	7/13 (54)	9/14 (64)	
aRNP titre; median (IQR)	26 (1.5–138)	105 (7-194.5)	*p* = 0.218
Low C3/C4; n (%)	5/9 (56)	11/12 (92)	
eGFR (mL/min/1.72m^2^); median (IQR)	98(81–115)	88(70-114.5)	*p* = 0.559
U-PCR (g/24 h); Median (IQR)	0.4(0.22–1.14)	1.34(0.33–4.79)	*p* = 0.137
CK (U/L); median (IQR)	57(26.25–132.5)	81(21.75–159.3)	*p* = 0.745
Troponin (ng/L); median (IQR)	4(2-6.75)	35.5(7.75–151)	*p* = 0.002
*Additional presentation*:			
Vasculitis; n (%)	1/13 (8)	1/14 (7)	
Arthritis; n (%)	2/13 (15)	7/14 (50)	
Pericarditis; n (%)	1/13 (8)	2/14 (14)	
Pleuritis; n (%)	1/13 (8)	4/14 (29)	
Lupus nephritis; n (%)	4/13 (31)	5/14 (36)	

Treatment exposure varied among the cohort as a whole. Twenty-two patients (81.5%) were on chloroquine, in nine patients this was initiated at the time of inclusion (range 0–20 days). A high dose corticosteroid (CS) (including prednisone and or intravenous methylprednisolone) was administered in 10/27 patients (37%) at a median of 4.5 days prior to inclusion. Additional immunosuppression (including cyclophosphamide (6/27) and mycophenolate mofetil (2/27)) was administered to a minority of patients prior to inclusion. To limit potential confounding effects of varied treatment regimes, CS exposure dose was statistically assessed for direct effects on serum cytokine levels. No significant differences in serum cytokine levels were detected in relation to high dose CS exposure (Supp Fig. 2). Further potential treatment effects were not statistically considered in data interpretation, as data was too limited. Nevertheless, in the absence of larger data sets (which currently do not exist for this population) which would allow for more in-depth consideration of specific treatment effects, the current data set was deemed best current representation of the total population.

### Distinct serum cytokine profile in lupus myocarditis compared to non-myocarditis SLE patients

**Fig. 1:**
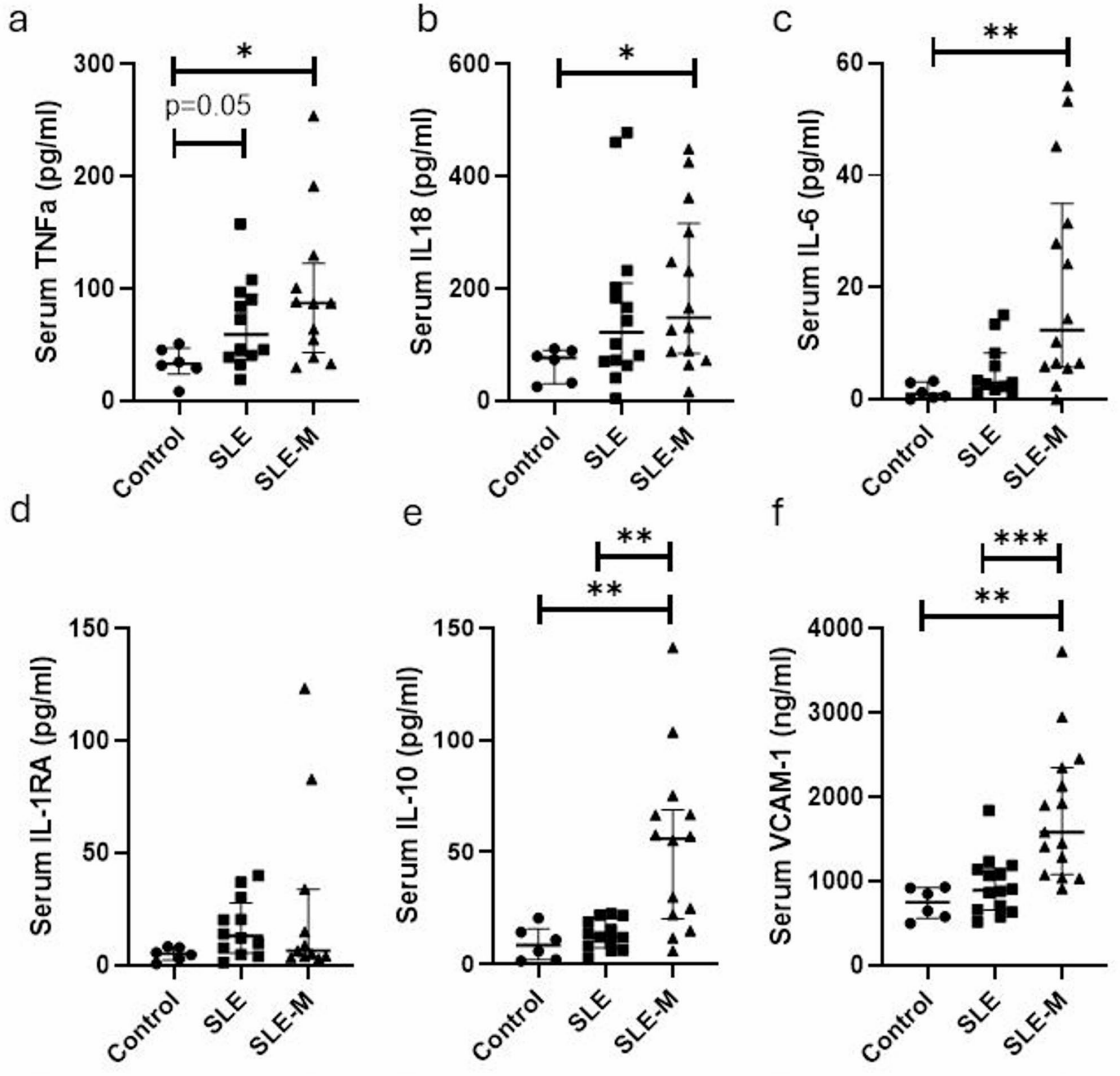
Comparison of serum cytokines between control, systemic lupus erythematosus (SLE) and lupus myocarditis patients (SLE-M). **a** TNF-α; (**b**) IL-18; (**c**) IL-6; (**d**) IL-1Ra; (**e**) IL-10; (**f**) VCAM-1. *n* = 6 healthy control, *n* = 13 SLE, *n* = 14 SLE-M. Data represented as median ± IQR. *,*p* < 0.05; **,*p* < 0.01; ***,*p* < 0.001. Tumor necrosis factor-α (TNF-α); Interleukin (IL), Vascular adhesion molecule-1 (VCAM-1). Data analysed with Kruskal-Wallis test

Analysis of serum cytokine markers (Fig. 1) demonstrated an increase in pro-inflammatory marker TNF-α levels in both SLE groups when compared to controls. When comparing SLE-M patients to controls, serum levels of pro-inflammatory TNF-α, as well as both IL-18 and, were significantly higher in SLE-M. Serum levels of anti-inflammatory IL-10 and adhesion molecule, VCAM-1, were significantly elevated in SLE-M when compared to both healthy controls and SLE patients. The large percentage change observed between SLE-M vs. SLE groups, (↑295%; *p* < 0.01 for IL-10 and ↑91%; *p* < 0.001 for VCAM-1 respectively), position both IL-10 and VCAM-1 as potential markers of interest in the distinction between SLE and SLE-M. In this context, VCAM-1 showed more uniform elevation in all SLE-M patients, while the IL-10 response appeared to exhibit responders and non-responders. While not reaching statistical significance due to high variability in the SLE-M group, average serum IL-6 increased by 290% in SLE-M compared to the SLE group.

Correlation analysis between serum cytokines (Table 2; Supp Fig. 3) demonstrated significant positive correlations between TNF-α and IL-18, IL-1Ra, and VCAM-1, IL-1Ra and IL-6, and IL-18 and VCAM-1 in the SLE-M group only.

**Table 2:** Comparison of serum (s) correlations between control, systemic lupus erythematosus (SLE) and lupus myocarditis (SLE-M) groups indicating the p-values, r-value and direction, and the 95% confidence interval (CI). Significance/tendency denoted in bold. Individual data points are presented in Fig. 2

	sIL-6	sIL-10	sTNF-α	sIL-18	sIL-1Ra
**Control**					
sIL-10	*p* = 0.658 *r* = 0.257 CI=-0.760 to 0.853				
sTNF-α	*p* = 0.175 *r* = 0.657 CI=-0.422 to 0.948	*p* = 0.803 *r* = 0.143 CI=-0.609 to 0.915			
sIL-18	*p* = 0.136 *r* = 0.714 CI=-0.356 to 0.955	*p* = 0.803 *r*=-0.143 CI=-0.803 to 0.820	*p* = 0.497 *r* = 0.371 CI=-0.414 to 0.949		
sIL-1Ra	*p* = 0.564 *r* = 0.314 CI=-0.635 to 0.908	*p* = 1.000 *r*=-0.029 CI=-0.798 to 0.825	*p* = 0.356 *r* = 0.486 CI=-0.420 to 0.948	*p* = 0.803 *r*=-0.143 CI=-0.784 to 0.836	
sVCAM-1	*p* = 0.919 *r* = 0.086 CI=-0.894 to 0.675	*p* = 0.175 *r* = 0.657 CI=-0.390 to 0.952	*p* = 0.658 *r* = 0.257 CI=-0.636 to 0.907	*p* = 0.919 *r* = 0.086 CI=-0.780 to 0.839	*p* = 0.919 *r* = 0.086 CI=-0.692 to 0.888
SLE					
sIL-10	*p* = 0.644 *r*=-0.183 CI=-0.725 to 0.593				
sTNF-α	*p* = 0.755 *r*=-0.109 CI=-0.677 to 0.540	*p* = 0.513 *r* = 0.236 CI=-0.528 to 0.713			
sIL-18	*p* = 0.818 *r*=-0.082 CI=-0.662 to 0.559	*p* = 0.193 *r*=-0.406 CI=-0,802 to 0.238	*p* = 0.128 *r*=-0.469 CI=-0.828 to 0.163		
sIL-1Ra	*p* = 0.973 *r* = 0.018 CI=-0.697 to 0.552	*p* = 0.468 *r* = 0.245 CI=-0.432 to 0.746	*p* = 0.279 *r*=-0.382 CI=-0.868 to 0.157	*p* = 0.155 *r* = 0.464 CI=-0.208 to 0.838	
sVCAM-1	*p* = 0.054 *r*=-0.636 **CI=-0.868 to 0.157**	*p* = 0.656 *r* = 0.137 CI=-0.462 to 0.651	*p* = 0.778 *r* = 0.482 CI=-0.186 to 0.845	*p* = 0.778 *r*=-0.088 CI=-0.621 to 0.501	*p* = 0.683 *r* = 0.133 CI=-0.492 to 0.668
SLE-M					
sIL-10	*p* = 0.964 *r*=-0.015 CI=-0.554 to 0.532				
sTNF-α	*p* = 0.904 *r* = 0.042 CI=-0.559 to 0.614	*p* = 0.276 *r* = 0.343 CI=-0.306 to 0.774			
sIL-18	*p* = 0.573 *r* = 0.165 CI=-0.415 to 0.650	*p* = 0.165 *r* = 0.393 CI=-0.190 to 0.772	*p* = 0.017 *r* = 0.685 **CI = 0.165 to 0.907**		
sIL-1Ra	*p* = 0.008 *r* = 0.833 **CI = 0.039 to 0.899**	*p* = 0.678 *r* = 0.167 CI=-0.440 to 0.742	*p* = 0.048 *r* = 0.786 **CI = 0.583 to 0.979**	*p* = 0.067 *r* = 0.650 CI=-0.062 to 0.878	
sVCAM-1	*p* = 0.671 *r* = 0.125 CI=-0.448 to 0.626	*p* = 0.532 *r* = 0.182 CI=-0.400 to 0.660	*p* = 0.006 *r* = 0.762 **CI = 0.318 to 0.932**	*p* = 0.032 *r* = 0.582 **CI = 0.058 to 0.855**	*p* = 0.213 *r* = 0.467 CI = 0.119 to 0.913

### Serum vs. tissue correlation analysis

**Fig. 2:**
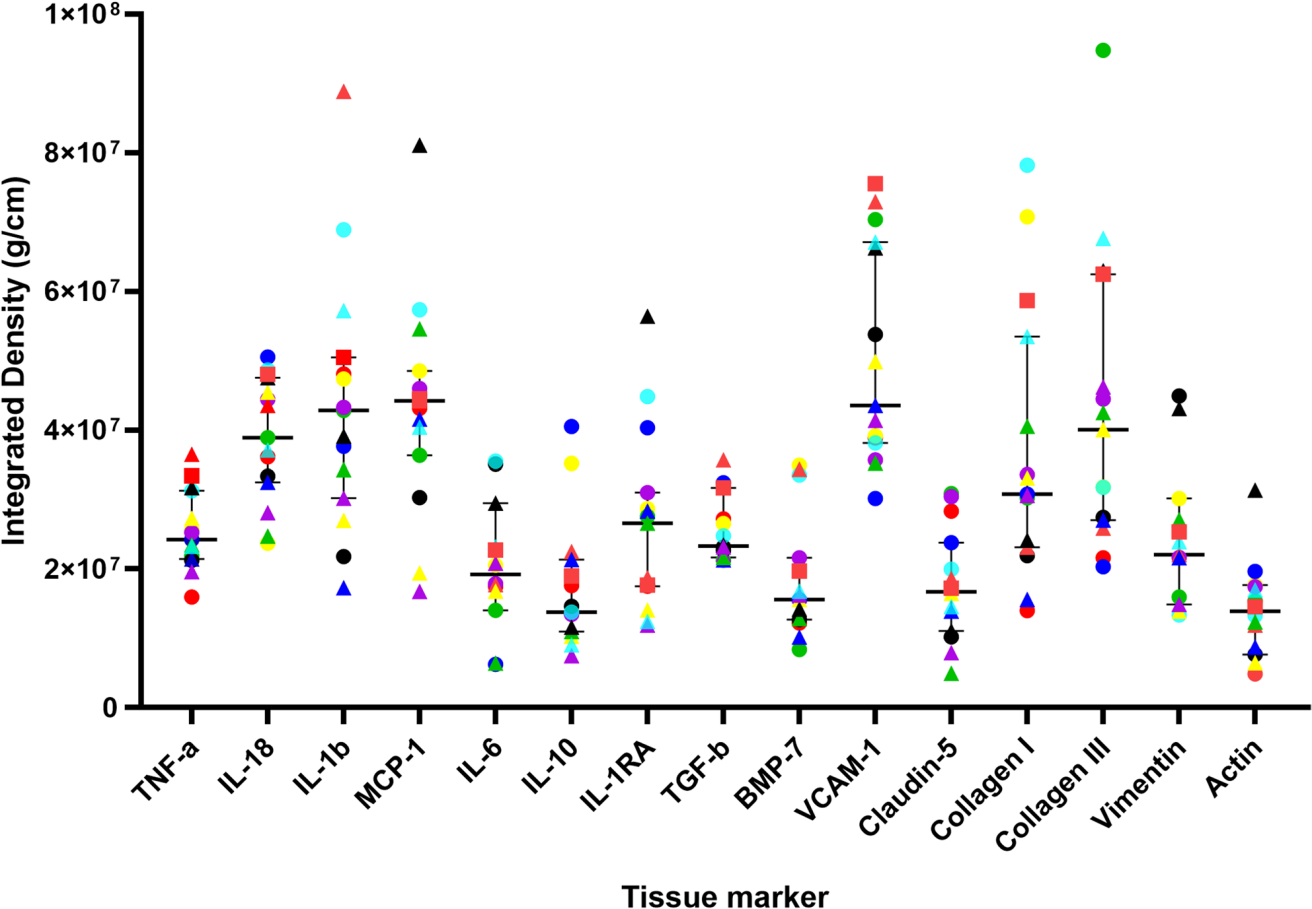
Illustration of variability and individual signalling profiles in myocardial biopsies of SLE myocarditis patients using immunofluorescence microscopy and measured using integrated density. Each icon represents a different patient (each patient is a designated icon throughout). Median (IQR) is presented

**Fig. 3:**
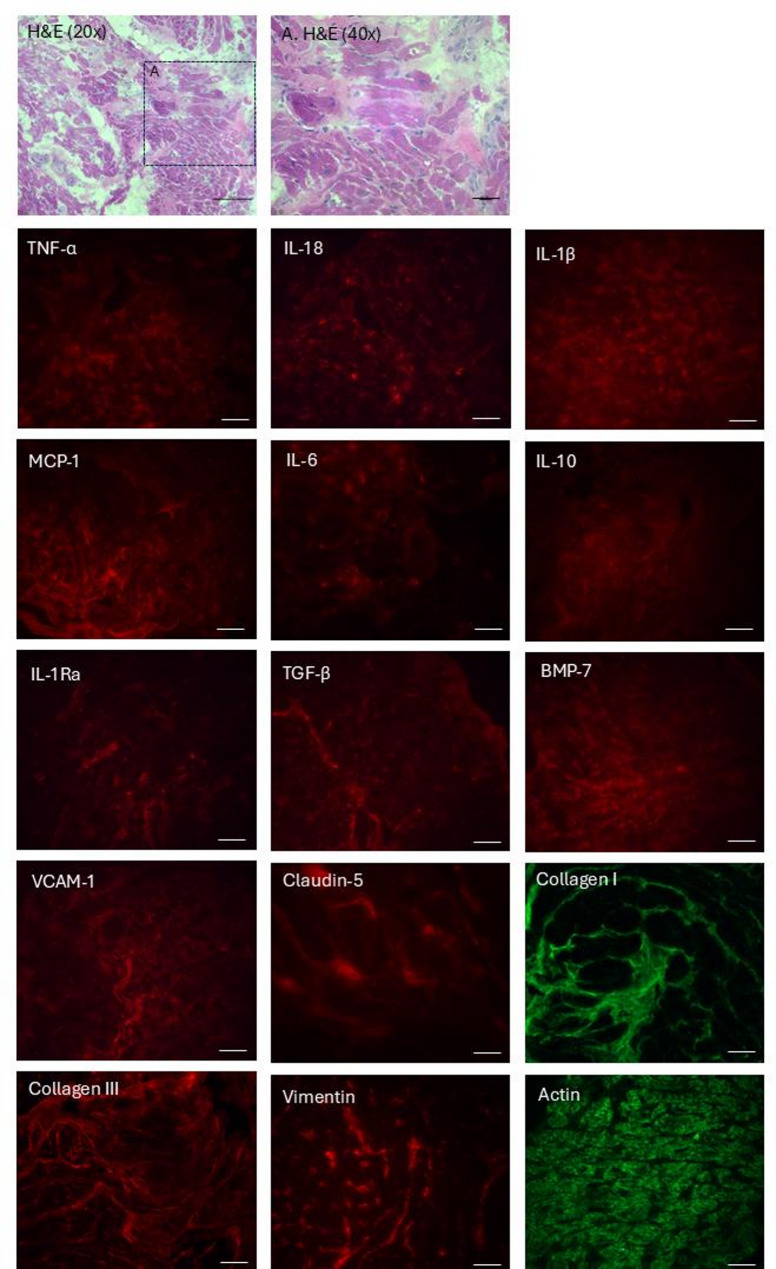
Representative images of brightfield haematoxylin and eosin staining (at 200x and 400x magnification) and immunofluorescent staining of tissue markers of inflammation, damage and fibrosis in SLE myocarditis endomyocardial biopsy tissue. Images taken at 400x magnification. Scale bar represents 100 μm (200x image) and 20 μm (400x images). Negative staining controls are demonstrated in Supp Fig. 1

With regards to cardiac tissue parameters, endomyocardial biopsy (EMB) tissue from the SLE-M group exhibited variable cytokine profiles (Figs. 2 and 3). Furthermore, it is of importance to note that tissue and serum levels of the same cytokines did not correlate (Table 3).

The comparison of serum vs. tissue profile does yield additional insights though (Table 3 and Supp Fig. 4). Contributing to the pro-inflammatory profile, serum IL-18 demonstrated a tendency for positive correlation with tissue IL-1β. Systemic inflammatory signalling via TNF-α, IL-1Ra, and VCAM-1 negatively correlated with tissue IL-6 expression. Serum IL-10 positively correlated to tissue MCP-1 as well as tissue actin. Additionally, serum IL-6 and IL-1Ra both positively correlated to tissue collagen III.

**Table 3:** Correlations between different serum cytokines with tissue cytokine in lupus myocarditis (SLE-M). Tissue presented on the left side of the table and serum presented on the top of the table. Table indicates p-values, r-values with direction, and 95% confidence intervals. Significance/tendency denoted in bold. s = serum; t = tissue. Individual data points are presented in Supp Fig. 4

	s-TNF-α	s-IL-18	s-IL-6	s-IL-10	s-IL-1Ra	s-VCAM-1
t-TNF-α	*p* = 0.558 *r* = 0.189 CI=-0.448 to 0.698	*p* = 0.227 *r* = 0.345 CI=-0.244 to 0.748	*p* = 0.762 *r*=-0.090 CI=-0.604 to 0.476	*p* = 0.160 *r* = 0.397 CI=-0.185 to 0.773	*p* = 0.468 *r* = 0.246 CI=-0.432 to 0.746	*p* = 0.667 *r*=-0.121 CI=-0.479 to 0.567
t-IL-18	*p* = 0.499 *r*=-0.217 CI=-0.713 to 0.424	*p* = 0.288 *r* = 0.306 CI=-0.285 to 0.728	*p* = 0.473 *r*=-0.209 CI=-0.675 to 0.377	*p* = 0.562 *r* = 0.169 CI=-0.412 to 0.652	*p* = 0.521 *r*=-0.218 CI=-0.733 to 0.456	*p* = 0.466 *r* = 0.204 CI=-0.359 to 0.658
t-IL-1β	*p* = 0.471 *r* = 0.231 CI=-0.412 to 0.720	*p* = 0.054 *r* = 0.530 **CI=-0.019 to 0.833**	*p* = 0.341 *r*=-0.275 CI=-0.712 to 0.315	*p* = 0.160 *r* = 0.398 CI=-0.185 to 0.774	*p* = 0.946 *r*=-0.027 CI=-0.630 to 0.596	*p* = 0.397 *r* = 0.236 CI=-0.330 to 0.677
t-MCP-1	*p* = 0.218 *r* = 0.385 CI=-0.261 to 0.793	*p* = 0.197 *r* = 0.367 CI=-0.220 to 0.759	*p* = 0.868 *r*=-0.051 CI=-0.578 to 0.506	*p* = 0.046 *r* = 0.547 **CI = 0.006 to 0.841**	*p* = 0.386 *r* = 0.291 CI=-0.392 to 0.767	*p* = 0.347 *r* = 0.261 CI=-0.306 to 0.691
t-IL-6	*p* = 0.016 *r*=-0.718 **CI=-0.924 to -0.188**	*p* = 0.224 *r*=-0.363 CI=-0.769 to 0.253	*p* = 0.835 *r*=-0.066 CI=-0.607 to 0.517	*p* = 0.863 *r* = 0.055 CI=-0.525 to 0.600	*p* = 0.046 *r*=-0.642 **CI=-0.906 to -0.0203**	*p* = 0.021 *r*=-0.608 **CI=-0.798 to 0.122**
t-IL-10	*p* = 0.266 *r* = 0.350 CI=-0.298 to 0.777	*p* = 0.203 *r* = 0.363 CI=-0.225 to 0.757	*p* = 0.160 *r*=-0.398 CI=-0.774 to 0.185	*p* = 0.418 *r* = 0.235 CI=-0.353 to 0.690	*p* = 0.451 *r*=-0.255 CI=-0.750 to 0.425	*p* = 0.934 *r* = 0.025 CI=-0.506 to 0.542
t-IL-1Ra	*p* = 0.573 *r* = 0.182 CI=-0.453 to 0.694	*p* = 0.295 *r* = 0.301 CI=-0.289 to 0.726	*p* = 0.693 *r*=-0.117 CI=-0.620 to 0.455	*p* = 0.067 *r* = 0.508 CI=-0.049 to 0.824	*p* = 0.435 *r* = 0.264 CI=-0.417 to 0.755	*p* > 0.999 *r* = 0.000 CI=-0.525 to 0.525
t-TGF-β	*p* = 0.766 *r* = 0.098 CI=-0.519 to 0.647	*p* = 0.091 *r* = 0.473 CI=-0.0948 to 0.808	*p* = 0.234 *r*=-0.341 CI=-0.746 to 0.248	*p* = 0.097 *r* = 0.464 CI=-0.106 to 0.804	*p* = 0.225 *r*=-0.400 CI=-0.813 to 0.282	*p* = 0.464 *r* = 0.213 CI=-0.373 to 0.678
t-BMP-7	*p* = 0.991 *r* = 0.007 CI=-0.582 to 0.591	*p* = 0.976 *r* = 0.011 CI=-0.535 to 0.551	*p* = 0.209 *r*=-0.358 CI=-0.755 to 0.229	*p* = 0.209 *r* = 0.358 CI=-0.229 to 0.755	*p* = 0.485 *r*=-0.236 CI=-0.742 to 0.440	*p* = 0.576 *r*=-0.157 CI=-0.630 to 0.400
t-VCAM-1	*p* = 0.558 *r*=-0.189 CI=-0.698 to 0.448	*p* = 0.616 *r* = 0.147 CI=-0.430 to 0.639	*p* = 0.318 *r* = 0.288 CI=-0.302 to 0.719	*p* = 0.856 *r*=-0.055 CI=-0.581 to 0.503	*p* = 0.818 *r* = 0.082 CI=-0.559 to 0.662	*p* = 0.832 *r* = 0.061 CI=-0.479 to 0.567
t-Claudin-5	*p* = 0.651 *r* = 0.147 CI=-0.481 to 0.675	*p* = 0.062 *r* = 0.517 CI=-0.037 to 0.827	*p* = 0.267 *r*=-0.319 CI=-0.735 to 0.271	*p* = 0.964 *r*=-0.015 CI=-0.532 to 0.554	*p* = 0.903 *r*=-0.045 CI=-0.640 to 0.584	*p* = 0.695 *r*=-0.111 CI=-0.439 to 0.600
t-Collagen I	*p* = 0.558 *r* = 0.189 CI=-0.448 to 0.698	*p* = 0.374 *r*=-0.257 CI=-0.702 to 0.332	*p* = 0.409 *r*=-0.240 CI=-0.693 to 0.349	*p* = 0.325 *r* = 0.284 CI=-0.307 to 0.716	*p* = 0.735 *r* = 0.118 CI=-0.533 to 0.682	*p* = 0.667 *r* = 0.121 CI=-0.431 to 0.607
t-Collagen III	*p* = 0.939 *r* = 0.028 CI=-0.568 to 0.605	*p* = 0.820 *r*=-0.068 CI=-0.589 to 0.493	*p* = 0.035 *r* = 0.574 **CI = 0.0445 to 0.851**	*p* = 0.832 *r* = 0.064 CI=-0.497 to 0.587	*p* = 0.028 *r* = 0.673 **CI = 0.102 to 0.910**	*p* = 0.397 *r* = 0.236 CI=-0.330 to 0.677
t-Vimentin	*p* = 0.107 *r* = 0.518 CI=-0.139 to 0.858	*p* = 0.457 *r* = 0.239 CI=-0.406 to 0.724	*p* = 0.817 *r* = 0.077 CI=-0.534 to 0.635	*p* = 0.939 *r* = 0.028 CI=-0.568 to 0.605	*p* = 0.248 *r* = 0.406 CI=-0.597 to 0.660	*p* = 0.081 *r* = 0.506 CI=-0.081 to 0.832
t-Actin	*p* = 0.088 *r* = 0.543 CI=-0.101 to 0.868	*p* = 0.216 *r* = 0.368 CI=-0.247 to 0.772	*p* = 0.643 *r* = 0.143 CI=-0.458 to 0.654	*p* = 0.014 *r* = 0.676 **CI = 0.181 to 0.898**	*p* = 0.088 *r* = 0.576 CI=-0.467 to 0.751	*p* = 0.194 *r* = 0.367 CI=-0.220 to 0.759

### Cardiac tissue correlations: pro and anti-inflammatory signalling

Within the cardiac tissue from SLE-M patients, correlations between assessed markers provide potential insight into the relationships between inflammation, fibrosis and remodelling (Table 4 & Supp Fig. 5). On the pro-inflammatory side, there are significant positive correlations between tissue TNF-α and both IL-18 and MCP-1, which would be expected in an inflammatory condition such as LM. Leukocyte chemoattraction (MCP-1) correlates positively with anti-inflammatory IL-1Ra while both markers also correlated positively to actin. In terms of vascular integrity and endothelial function, tissue IL-1β positively correlated to claudin-5.

Persistent inflammation in the tissue in the form of TNF-α and IL-1β correlated with an increase in BMP-7, which also in turn correlated with TGF-β. BMP-7 positively correlated with collagen I, the more structural collagen. However, in addition to the positive correlation between IL-10 and TGF-β, the higher levels of IL-10 correlated with a decrease in collagen III - a more elastic form of collagen.

To note is the lack of any significant correlation between tissue IL-6, and both pro-inflammatory and anti-inflammatory parameters. Similarly, tissue IL-10 levels failed to show any correlation with early pro-inflammatory markers.

**Table 4:** Correlations between different tissue parameters (t) in lupus myocarditis (SLE-M). Table indicates p-values, r-values with direction and 95% confidence intervals (CI). Significance denoted in bold. Individual data points are presented in Supp Fig. 5

	t-TNF-α	t-IL-18	t-IL-1β	t-MCP-1	t-IL-6	t-IL-10	t-IL-1Ra	t-TGF-β	t-BMP-7	t-VCAM-1	t-Claudin-5	t-Collagen I	t-Collagen III	t-Vimentin
t-IL-18	*p* = 0.027 *r* = 0.575 CI = 0.072 to 0.845													
t-IL-1β	*p* = 0.069 *r* = 0.486 CI=-0.052 to 0.805	*p* = 0.237 *r* = 0.325 CI=-0.241 to 0.726												
t-MCP-1	*p* = 0.038 *r* = 0.546 **CI = 0.031 to 0.832**	*p* = 0.327 *r* = 0.271 CI=-0.295 to 0.697	*p* = 0.153 *r* = 0.389 CI=-0.170 to 0.759											
t-IL-6	*p* = 0.542 *r* = 0.178 CI=-0.404 to 0.658	*p* = 0.832 *r* = 0.064 CI=-0.497 to 0.587	*p* = 0.427 *r* = 0.231 CI=-0.357 to 0.688	*p* = 0.671 *r* = 0.125 CI=-0.448 to 0.626										
t-IL-10	*p* = 0.450 *r* = 0.211 CI=-0.353 to 0.662	*p* = 0.630 *r* = 0.136 CI=-0.419 to 0.616	*p* = 0.498 *r* = 0.189 CI=-0.372 to 0.649	*p* = 0.301 *r* = 0.286 CI=-0.281 to 0.705	*p* = 0.605 *r*=-0.152 CI=-0.642 to 0.427									
t-IL-1Ra	*p* = 0.149 *r* = 0.393 CI=-0.166 to 0.761	*p* = 0.182 *r* = 0.364 CI=-0.198 to 0.746	*p* = 0.812 *r* = 0.068 CI=-0.474 to 0.572	*p* = 0.001 *r* = 0.782 **CI = 0.437 to 0.927**	*p* = 0.916 *r* = 0.033 CI=-0.519 to 0.566	*p* = 0.123 *r* = 0.418 CI=-0.137 to 0.773								
t-TGF-β	*p* = 0.120 *r* = 0.437 CI=-0.139 to 0.792	*p* = 0.151 *r* = 0.407 CI=-0.175 to 0.778	*p* = 0.010 *r* = 0.675 **CI = 0.208 to 0.891**	*p* = 0.295 *r* = 0.301 CI=-0.289 to 0.726	*p* = 0.696 *r* = 0.121 CI=-0.475 to 0.641	*p* = 0.038 *r* = 0.565 **CI = 0.0315 to 0.848**	*p* = 0.820 *r* = 0.068 CI=-0.493 to 0.589							
t-BMP-7	*p* = 0.011 *r* = 0.646 **CI = 0.185 to 0.874**	*p* = 0.507 *r* = 0.186 CI=-0.375 to 0.647	*p* = 0.017 *r* = 0.614 **CI = 0.133 to 0.861**	*p* = 0.221 *r* = 0.336 CI=-0.229 to 0.731	*p* = 0.267 *r* = 0.319 CI=-0.271 to 0.735	*p* = 0.667 *r* = 0.121 CI=-0.431 to 0.607	*p* = 0.773 *r* = 0.082 CI=-0.462 to 0.582	*p* = 0.027 *r* = 0.596 **CI = 0.078 to 0.860**						
t-VCAM-1	*p* = 0.320 *r* = 0.275 CI=-0.292 to 0.699	*p* = 0.763 *r* = 0.086 CI=-0.459 to 0.584	*p* = 0.411 *r* = 0.229 CI=-0.336 to 0.672	*p* = 0.187 *r*=-0.361 CI=-0.744 to 0.202	*p* = 0.253 *r* = 0.327 CI=-0.262 to 0.739	*p* = 0.842 *r*=-0.057 CI=-0.565 to 0.482	*p* = 0.192 *r*=-0.357 CI=-0.743 to 0.206	*p* = 0.988 *r* = 0.007 CI=-0.538 to 0.548	*p* = 0.995 *r* = 0.004 CI=-0.522 to 0.527					
t-Claudin-5	*p* = 0.584 *r*=-0.154 CI=-0.403 to 0.628	*p* = 0.067 *r* = 0.489 CI=-0.047 to 0.807	*p* = 0.052 *r* = 0.514 **CI=-0.014 to 0.818**	*p* = 0.667 *r* = 0.121 CI=-0.431 to 0.607	*p* = 0.302 *r*=-0.297 CI=-0.723 to 0.294	*p* = 0.133 *r* = 0.407 CI=-0.149 to 0.768	*p* = 0.307 *r* = 0.282 CI=-0.284 to 0.703	*p* = 0.366 *r* = 0.262 CI=-0.328 to 0.705	*p* = 0.676 *r* = 0.118 CI=-0.433 to 0.605	*p* = 0.863 *r*=-0.050 CI=-0.559 to 0.487				
t-Collagen I	*p* = 0.081 *r* = 0.468 CI=-0.075 to 0.797	*p* = 0.507 *r* = 0.186 CI=-0.375 to 0.647	*p* = 0.141 *r* = 0.400 CI=-0.158 to 0.764	*p* = 0.141 *r* = 0.400 CI=-0.158 to 0.764	*p* = 0.532 *r* = 0.182 CI=-0.400 to 0.660	*p* = 0.621 *r*=-0.139 CI=-0.619 to 0.416	*p* = 0.630 *r* = 0.136 CI=-0.419 to 0.616	*p* = 0.693 *r* = 0.116 CI=-0.455 to 0.620	*p* = 0.009 *r* = 0.661 **CI = 0.209 to 0.880**	*p* = 0.549 *r*=-0.168 CI=-0.636 to 0.391	*p* = 0.8883 *r*=-0.043 CI=-0.493 to 0.555			
t-Collagen III	*p* = 0.734 *r* = 0.096 CI=-0.451 to 0.591	*p* = 0.954 *r* = 0.186 CI=-0.537 to 0.511	*p* = 0.793 *r* = 0.075 CI=-0.468 to 0.577	*p* = 0.822 *r*=-0.064 CI=-0.570 to 0.476	*p* = 0.473 *r* = 0.209 CI=-0.377 to 0.675	*p* = 0.008 *r*=-0.664 **CI=-0.882 to -0.215**	*p* = 0.593 *r*=-0.150 CI=-0.625 to 0.407	*p* = 0.137 *r*=-0.420 CI=-0.784 to 0.160	*p* = 0.995 *r*=-0.004 CI=-0.527 to 0.522	*p* = 0.100 *r* = 0.443 CI=-0.106 to 0.785	*p* = 0.621 *r*=-0.139 CI=-0.619 to 0.416	*p* = 0.192 *r* = 0.357 CI=-0.206 to 0.743		
t-Vimentin	*p* = 0.835 *r* = 0.066 CI=-0.517 to 0.607	*p* = 0.334 *r*=-0.018 CI=-0.734 to 0.326	*p* = 0.863 *r*=-0.055 CI=-0.600 to 0.525	*p* = 0.289 *r* = 0.319 CI=-0.299 to 0.748	*p* = 0.558 *r* = 0.189 CI=-0.448 to 0.698	*p* = 0.373 *r* = 0.269 CI=-0.347 to 0.723	*p* = 0.696 *r* = 0.121 CI=-0.475 to 0.641	*p* = 0.543 *r* = 0.196 CI=-0.442 to 0.702	*p* = 0.892 *r*=-0.044 CI=-0.593 to 0.532	*p* = 0.617 *r* = 0.154 CI=-0.449 to 0.660	*p* = 0.176 *r*=-0.401 CI=-0.787 to 0.210	*p* = 0.683 *r*=-0.126 CI=-0.644 to 0.471	*p* = 0.935 *r*=-0.027 CI=-0.545 to 0.582	
t-Actin	*p* = 0.221 *r* = 0.349 CI=-0.238 to 0.750	*p* = 0.281 *r* = 0.310 CI=-0.280 to 0.730	*p* = 0.553 *r* = 0.174 CI=-0.407 to 0.654	*p* = 0.035 *r* = 0.574 **CI = 0.365 to 0.916**	*p* = 0.806 *r* = 0.077 CI=-0.509 to 0.614	*p* = 0.844 *r* = 0.059 CI=-0.500 to 0.584	*p* = 0.021 *r* = 0.618 **CI = 0.311 to 0.906**	*p* = 0.541 *r* = 0.187 CI=-0.421 to 0.679	*p* = 0.260 *r* = 0.323 CI=-0.267 to 0.737	*p* = 0.785 *r*=-0.081 CI=-0.598 to 0.483	*p* = 0.638 *r*=-0.138 CI=-0.437 to 0.634	*p* = 0.116 *r* = 0.442 CI=-0.133 to 0.794	*p* = 0.155 *r* = 0.402 CI=-0.180 to 0.776	*p* = 0.366 *r* = 0.287 CI=-0.361 to 0.748

### Correlations between tissue and clinical disease parameters

Next, we considered relationships between serum markers of clinical disease and tissue assessments in the SLE-M group only (Table 5 and Supp Fig. 6). Inflammatory activation in the cardiac tissue corresponded with the presence of certain autoantibodies as noted by correlations between aRo and both tissue IL-1β and TGF-β, between aLa and tissue TGF-β as well as between anti-dsDNA and tissue IL-1β, MCP-1 and IL-1Ra. aRo was also associated with tight junctions (claudin-5). In agreement with the profile in the tissue described above, increasing aLa titre also paralleled increasing tissue BMP-7 abundance. CK negatively correlated to tissue VCAM-1 while troponin levels did not correlate with any tissue parameters.

**Table 5:** Correlations between clinical (standard of care) markers and tissue parameters in lupus myocarditis (SLE-M) patients. Table indicates p-values, r-values with direction, and 95% confidence intervals (CI). Significance/tendency denoted in bold. Individual data points are presented in Supp Fig. 6

	aRo	aLa	Anti-dsDNA	CK	Troponin	eGFR	U-PCR
t-TNF-α	*p* = 0.818 *r* = 0.081 CI=-0.561 to 0.636	*p* = 0.209 *r* = 0.503 CI=-0.062 to 0.934	*p* = 0.103 *r* = 0.456 CI=-0.116 to 0.801	*p* = 0.854 *r* = 0.075 CI=-0.620 to 0.703	*p* = 0.164 *r*=-0.455 CI=-0.834 to 0.219	*p* = 0.723 *r*=-0.110 CI=-0.634 to 0.484	*p* = 0.858 *r*=-0.053 CI=-0.506 to 0.577
t-IL-18	*p* = 0.299 *r* = 0.346 CI=-0.171 to 0.838	*p* = 0.317 *r* = 0.407 CI=-0.131 to 0.925	*p* = 0.471 *r* = 0.209 CI=-0.376 to 0.675	*p* = 0.528 *r* = 0.243 CI=-0.659 to 0.670	*p* = 0.796 *r*=-0.091 CI=-0.667 to 0.553	*p* = 0.935 *r*=-0.027 CI=-0.582 to 0.545	*p* = 0.337 *r*=-0.278 CI=-0.670 to 0.336
t-IL-1β	*p* = 0.040 *r* = 0.636 **CI=-0.292 to 0.795**	*p* = 0.125 *r* = 0.599 CI=-0.127 to 0.925	*p* = 0.038 *r* = 0.565 **CI=**-**0.031 to 0.848**	*p* = 0.327 *r* = 0.368 CI=-0.511 to 0.776	*p* = 0.342 *r* = 0.318 CI=-0.366 to 0.779	*p* = 0.696 *r*=-0.121 CI=-0.641 to 0.475	*p* = 0.384 *r* = 0.252 CI=-0.385 to 0.670
t-MCP-1	*p* = 0.356 *r* = 0.309 CI=-0.393 to 0.749	*p* = 0.471 *r* = 0.299 CI=-0.400 to 0.869	*p* = 0.021 *r* = 0.616 **CI = 0.109 to 0.868**	*p* = 0.150 *r* = 0.527 CI=-0.134 to 0.899	*p* = 0.990 *r*=-0.009 CI=-0.619 to 0.607	*p* = 0.036 *r*=-0.593 **CI=-0.867 to -0.045**	*p* = 0.300 *r*=-0.298 CI=-0.497 to 0.587
t-IL-6	*p* = 0.179 *r*=-0.467 CI=-0.870 to 0.146	*p* = 0.859 *r* = 0.090 CI=-0.640 to 0.834	*p* = 0.872 *r*=-0.050 CI=-0.596 to 0.525	*p* = 0.548 *r*=-0.252 CI=-0.871 to 0.392	*p* = 0.946 *r*=-0.030 CI=-0.829 to 0.288	*p* = 0.309 *r*=-0.322 CI=-0.764 to 0.327	*p* = 0.873 *r*=-0.049 CI=-0.501 to 0.621
t-IL-10	*p* = 0.371 *r* = 0.300 CI=-0.383 to 0.771	*p* = 0.630 *r*=-0.204 CI=-0.740 to 0.666	*p* = 0.083 *r* = 0.482 CI=-0.082 to 0.812	*p* = 0.752 *r* = 0.126 CI=-0.689 to 0.638	*p* = 0.299 *r*=-0.346 CI=-0.791 to 0.339	*p* = 0.751 *r* = 0.099 CI=-0.492 to 0.628	*p* = 0.952 *r*=-0.042 CI=-0.572 to 0.513
t-IL-1Ra	*p* = 0.818 *r* = 0.081 CI=-0.408 to 0.741	*p* = 0.381 *r* = 0.359 CI=-0.014 to 0.940	*p* = 0.054 *r* = 0.529 **CI=-0.020 to 0.833**	*p* = 0.340 *r* = 0.360 CI=-0.388 to 0.830	*p* = 0.342 *r*=-0.318 CI=-0.779 to 0.366	*p* = 0.334 *r*=-0.291 CI=-0.734 to 0.326	*p* = 0.178 *r*=-0.382 CI=-0.652 to 0.411
t-TGF-β	*p* = 0.013 *r* = 0.770 **CI = 0.188 to 0.924**	*p* = 0.024 *r* = 0.845 **CI = 0.195 to 0.973**	*p* = 0.144 *r* = 0.429 CI=-0.177 to 0.799	*p* = 0.752 *r* = 0.143 CI=-0.621 to 0.772	*p* = 0.448 *r* = 0.273 CI=-0.531 to 0.711	*p* = 0.443 *r*=-0.245 CI=-0.770 to 0.399	*p* = 0.594 *r* = 0.156 CI=-0.158 to 0.806
t-BMP-7	*p* = 0.402 *r* = 0.282 CI=-0.400 to 0.762	*p* = 0.036 *r* = 0.755 **CI = 0.458 to 0.978**	*p* = 0.346 *r* = 0.271 CI=-0.319 to 0.710	*p* = 0.653 *r*=-0.176 CI=-0.742 to 0.568	*p* = 0.881 *r*=-0.055 CI=-0.646 to 0.577	*p* = 0.821 *r*=-0.071 CI=-0.611 to 0.513	*p* = 0.260 *r* = 0.323 CI=-0.267 to 0.737
t-VCAM-1	*p* = 0.371 *r*=-0.300 CI=-0.771 to 0.383	*p* = 0.100 *r*=-0.635 CI=-0.912 to 0.211	*p* = 0.745 *r*=-0.096 CI=-0.607 to 0.472	*p* = 0.040 *r*=-0.703 **CI=-0.828 to 0.397**	*p* = 0.714 *r*=-0.127 CI=-0.687 to 0.527	*p* = 0.176 *r* = 0.401 CI=-0.210 to 0.787	*p* = 0.856 *r*=-0.055 CI=-0.581 to 0.503
t-Claudin-5	*p* = 0.052 *r* = 0.609 **CI = 0.105 to 0.903**	*p* = 0.334 *r* = 0.395 CI=-0.014 to 0.940	*p* = 0.478 *r* = 0.207 CI=-0.364 to 0.665	*p* = 0.922 *r*=-0.042 CI=-0.754 to 0.549	*p* = 0.356 *r*=-0.309 CI=-0.775 to 0.375	*p* = 0.289 *r* = 0.319 CI=-0.299 to 0.748	*p* = 0.888 *r*=-0.042 CI=-0.584 to 0.499
t-Collagen I	*p* = 0.946 *r*=-0.273 CI=-0.759 to 0.373	*p* = 0.209 *r* = 0.503 CI=-0.207 to 0.913	*p* = 0.367 *r* = 0.260 CI=-0.330 to 0.704	*p* = 0.572 *r* = 0.218 CI=-0.604 to 0.717	*p* = 0.860 *r* = 0.064 CI=-0.572 to 0.651	*p* = 0.493 *r*=-0.209 CI=-0.691 to 0.402	*p* = 0.534 *r*=-0.182 CI=-0.665 to 0.393
t-Collagen III	*p* = 0.402 *r*=-0.282 CI=-0.840 to 0.162	*p* = 0.591 *r* = 0.228 CI=-0.748 to 0.656	*p* = 0.490 *r*=-0.200 CI=-0.670 to 0.385	*p* = 0.904 *r*=-0.050 CI=-0.658 to 0.671	*p* = 0.371 *r* = 0.300 CI=-0.383 to 0.771	*p* = 0.949 *r* = 0.022 CI=-0.548 to 0.578	*p* = 0.476 *r*=-0.208 CI=-0.595 to 0.486
t-Vimentin	*p* = 0.912 *r* = 0.050 CI=-0.186 to 0.845	*p* = 0.223 *r*=-0.491 CI=-0.224 to 0.834	*p* = 0.649 *r* = 0.148 CI=-0.481 to 0.676	*p* = 0.849 *r* = 0.084 CI=-0.554 to 0.811	*p* = 0.410 *r* = 0.317 CI=-0.652 to 0.676	*p* = 0.076 *r*=-0.564 CI=-0.874 to 0.075	*p* = 0.820 *r* = 0.068 CI=-0.470 to 0.683
t-Actin	*p* = 0.263 *r* = 0.394 CI=-0.339 to 0.791	*p* = 0.512 *r* = 0.306 CI=-0.412 to 0.909	*p* = 0.293 *r* = 0.315 CI=-0.303 to 0.746	*p* = 0.471 *r* = 0.299 CI=-0.544 to 0.816	*p* = 0.584 *r* = 0.200 CI=-0.355 to 0.804	*p* = 0.588 *r*=-0.175 CI=-0.691 to 0.459	*p* = 0.727 *r* = 0.103 CI=-0.641 to 0.475

### Correlations between tissue parameters and CMR

Assessment of the correlations between tissue parameters and CMR findings (Table 6 & Supp Fig. 7) demonstrated positive correlations between STIR vs. tissue IL-10 and tissue BMP-7. Higher T1 and T2 correlated with lower tissue IL-6 levels. Patients with detectable LGE, exhibited significantly higher tissue VCAM-1 vs. those of patients without detectable presence of LGE. However, none of the CMR markers correlated with tissue markers of fibrosis.

**Table 6:** Correlations between tissue parameters and CMR findings. Table indicates p-values, r-values with direction, and 95% confidence intervals (CI) (STIR, T2, T1); or p-values with median ± IQR and percentage change of the mean (LGE*) where relevant. Significance/tendency denoted in bold. *See below for description. Individual data points presented in Supp Fig. 7

	STIR	T2	T1	LGE
				*n* = 7(1); *n* = 5(2) (all values x10^6^)
t-TNF-α	*p* = 0.748 *r* = 0.104 CI=-0.514 to 0.651	*p* = 0.271 *r*=-0.328 CI=-0.753 to 0.289	*p* = 0.216 *r*=-0.368 CI=-0.772 to 0.247	*p* = 0.343 (1)25.65 ± 15.23 (2)24.27 ± 11.36
t-IL-18	*p* = 0.995 *r* = 0.004 CI=-0.584 to 0.589	*p* = 0.702 *r*=-0.117 CI=-0.638 to 0.478	*p* = 0.505 *r*=-0.203 CI=-0.688 to 0.407	*p* = 0.755 (1)38.92 ± 24.96 (2)44.45 ± 18.05
t-IL-1β	*p* = 0.097 *r* = 0.504 CI=-0.118 to 0.842	*p* = 0.172 *r*=-0.403 CI=-0.788 to 0.208	*p* = 0.920 *r*=-0.033 CI=-0.586 to 0.541	*p* = 0.202 (1)47.38 ± 67.05 (↑51%) (2)37.65 ± 30.77
t-MCP-1	*p* = 0.852 *r*=-0.061 CI=-0.625 to 0.545	*p* = 0.655 *r*=-0.136 CI=-0.650 to 0.463	*p* = 0.978 *r* = 0.011 CI=-0.556 to 0.571	*p* = 0.639 (1)44.20 ± 50.76 (2)43.16 ± 26.55
t-IL-6	*p* = 0.256 *r* = 0.373 CI=-0.311 to 0.802	*p* = 0.007 *r*=-0.749 **CI=-0.928 to -0.290**	*p* = 0.043 *r*=-0.601 **CI=-0.878 to -0.023**	*p* = 0.072 (1)23.22 ± 21.48 (↑72%) (2)17.15 ± 11.63
t-IL-10	*p* = 0.046 *r* = 0.593 **CI = 0.010 to 0.875**	*p* = 0.945 *r* = 0.022 CI=-0.548 to 0.579	*p* = 0.920 *r* = 0.033 CI=-0.541 to 0.586	*p* = 0.755 (1)13.78 ± 26.16 (2)17.63 ± 30.17
t-IL-1Ra	*p* = 0.968 *r*=-0.014 CI=-0.596 to 0.577	*p* = 0.923 *r*=-0.031 CI=-0.584 to 0.542	*p* = 0.737 *r*=-0.104 CI=-0.631 to 0.488	*p* = 0.876 (1)27.61 ± 43.95 (2)28.36 ± 26.23
t-TGF-β	*p* = 0.097 *r* = 0.504 CI=-0.118 to 0.842	*p* = 0.271 *r*=-0.328 CI=-0.753 to 0.289	*p* = 0.921 *r*=-0.033 CI=-0.586 to 0.541	*p* = 0.639 (1)24.81 ± 19.12 (2)23.19 ± 11.15
t-BMP-7	*p* = 0.052 *r* = 0.579 **CI=-0.0121 to 0.870**	*p* = 0.107 *r*=-0.470 CI=-0.817 to 0.127	*p* = 0.061 *r*=-0.539 CI=-0.846 to 0.036	*p* = 0.432 (1)16.82 ± 26.56 (2)15.57 ± 11.46
t-VCAM-1	*p* = 0.987 *r*=-0.007 CI=-0.582 to 0.591	*p* = 0.434 *r*=-0.237 CI=-0.706 to 0.378	*p* = 0.891 *r* = 0.044 CI=-0.533 to 0.593	*p* = 0.048 **(1)66.26 ± 34.75** **(↑47%)** **(2)38.84 ± 19.68**
t-Claudin-5	*p* = 0.218 *r* = 0.382 CI=-0.264 to 0.792	*p* = 0.696 *r* = 0.120 CI=-0.476 to 0.640	*p* = 0.793 *r* = 0.082 CI=-0.505 to 0.617	*p* = 0.432 (1)16.72 ± 20.70 (2)23.82 ± 16.52
t-Collagen I	*p* = 0.544 *r* = 0.193 CI=-0.444 to 0.703	*p* = 0.865 *r*=-0.053 CI=-0.599 to 0.526	*p* = 0.448 *r*=-0.231 CI=-0.703 to 0.383	*p* = 0.155 (1)30.27 ± 56.28 (↑70%) (2)30.81 ± 19.64
t-Collagen III	*p* = 0.120 *r*=-0.475 CI=-0.830 to 0.155	*p* = 0.709 *r* = 0.114 CI=-0.480 to 0.637	*p* = 0.765 *r* = 0.093 CI=-0.496 to 0.624	*p* = 0.182 (1)31.84 ± 68.83 (↑59%) (2)27.07 ± 24.12
t-Vimentin	*p* = 0.861 *r* = 0.057 CI=-0.548 to 0.623	*p* = 0.235 *r* = 0.353 CI=-0.263 to 0.765	*p* = 0.164 *r* = 0.412 CI=-0.197 to 0.792	*p* = 0.876 (1)23.96 ± 31.53 (2)21.66 ± 53.26
t-Actin	*p* = 0.879 *r*=-0.050 CI=-0.619 to 0.553	*p* = 0.688 *r* = 0.122 CI=-0.474 to 0.622	*p* = 0.710 *r* = 0.115 CI=-0.479 to 0.638	*p* = 0.271 (1)15.40 ± 23.69 (2)8.77 ± 14.80

*LGE was measured as presence (1) or absence (2). The table presents the mean ± SD integrated density (g/cm) in the presence of LGE signal (1) or the absence (2) as well as the p-value following t-test analysis. Where larger differences were noted, the percentage change (%) was also noted

## Discussion

Current data present identifying associations between various cytokines and standard of care measurements allowing the generation of hypotheses into potential mechanisms at play in the aetiology of myocarditis in SLE. Firstly, the apparent disconnect between inflammatory cytokine abundance in circulation and in cardiac tissue illustrated how monitoring of the circulating inflammatory profile does not inform on potential cumulative progressive inflammatory response at tissue level. Secondly, despite the limitation of not having absolute tissue cytokine concentrations, we hypothesise that relative lack of an expected IL-6 and IL-10 presence compared to early pro-inflammatory markers in cardiac tissue as well as CMR profile, may suggest a relative insufficiency of IL-6, and thus also IL-10, in the myocardium. Thirdly, we hypothesise that a resultant failure of tissue IL-6-activated endogenous anti-inflammatory modalities, may underpin progression of myocardial injury and fibrosis in LM.

In terms of the study population, data on serum cytokine profiles align with existing SLE and LM literature, illustrating a robust and reproducible cytokine signature in SLE, despite the relatively small cohort sizes reported in most studies. However, a number of serum cytokines, including TNF-α, IL-18, IL-6, IL-10 and VCAM-1, demonstrated increased abundance in the SLE-M group when compared to controls. Serum markers that appear to be of particular relevance in LM include IL-10 and VCAM-1, as demonstrated by the robust differences between the SLE-M group and both the controls and the SLE group. These results are in agreement with the literature, including that of a similar demographic group (Chen et al. 2014; Du Toit et al. 2017, 2021). Serum IL-10 has previously been suggested as a marker of disease activity in SLE due to a strong positive correlation with SLEDAI-2k scores (Godsell et al. 2016; Verma et al. 2024) and is noticeably elevated in LM patients in the current study. While most commonly known for its suppressive effect on pro-inflammatory cells, evidence indicates a dual role, also driving B cell responses and autoantibody production (Biswas et al. 2022). This would be fitting of its relationship to disease activity and its abundance in the serum in LM. Serum VCAM-1 has also previously been highlighted as a potential marker of LM due to its role in endothelial activation (Du Toit et al. 2021). Several studies in SLE have highlighted the role of endothelial activation in systemic organ damage, with several markers elevated and related to disease activity (Kuryliszyn-Moskal et al. 2008; Li et al. 2023). Overall, these results validate the patient population.

Determining correlations between various serum markers assists in delineating the inflammatory profile associated with LM. A significant positive correlation between serum VCAM-1 and TNF-α in SLE-M, which was not noted in controls, validates the relationship between inflammation and endothelial activation in LM (Kuryliszyn-Moskal et al. 2008). The relationship between TNF-α and IL-18 is also key in inflammatory conditions, with TNF-α inducing the synthesis and release of IL-18 from the inflammasome, and IL-18 further prolonging TNF-α signalling (Silliman et al. 2010). IL-1Ra is known to be a key role player in sustained inflammation in SLE and has been noted for its role in the development of myocarditis (Moosig et al. 2004; Du Toit et al. 2021). The results presented in the current study support this correlation between IL-1Ra and several other proinflammatory cytokines (including TNF-α, IL-6 and VCAM-1) in the serum of SLE patients. The greater number of correlations noted in the SLE-M group may indicate a robust inflammatory response which superseded the expected dampening effect of concomitant immunosuppressive therapy. Furthermore, despite more patients in the SLE-M group receiving corticosteroids, there was no significant difference in serum cytokines irrespective of treatment (*p* > 0.05; Supp Fig. 2), further supporting this interpretation.

To our knowledge, this is the first study to comprehensively profile cytokines, growth factors and fibrosis markers within the myocardial tissue of patients with LM, adding substantially more information to a previous report demonstrating similar expression patterns for TNF-α in the myocardial tissue in a case study of fatal cardiac failure in SLE (Pomara et al. 2010). Current data aligns with data from an early seminal paper, which suggested a major role for myocardial immune complexes in lethal cardiac injury in SLE (Bidani et al. 1980), and with the benefit or more advanced immunostaining and imaging methodology, contribute more mechanistic hypotheses. Given the absence of a suitable control tissue sample, interpretations on absolute cytokine abundance in this compartment is not possible. However, the presence or absence of correlations between different role players (and compartments) in inflammation, provides insights into potential sites of dysregulation at tissue level, which may be highlighted for further investigations and eventually exploited as therapeutic targets. In this context, it is of importance firstly that no direct correlations were observed between serum and tissue levels of cytokines. This suggests a potential disconnect between systemic and tissue level responses. Such a disparity is consistent with the entity of subclinical myocarditis in SLE, where clinical findings, serological markers, and myocardial pathology is typically discordant (Guo et al. 2018; Panchal et al. 2006). This has also been noted in other organs that contribute to mortality in SLE. In lupus nephritis, persistent renal inflammation on biopsy occurs in up to 50% of patients who achieved clinical remission, providing arguments towards histological confirmation of remission (Yo et al. 2019). Current findings support a serum cytokine profile that represents a current picture of global systemic inflammatory events, without necessarily reflecting the nett outcome of inflammation within the tissue itself. Due to the vast organ involvement in SLE, multiple sites of inflammation contribute to nett outcome reflected in serum, and as such, it should not be unexpected for serum and tissue profiles to differ.

Functionally contradictory negative correlations were observed between serum TNF-α vs. tissue IL-6 and between serum VCAM-1 vs. tissue IL-6, as well as a positive correlation between serum IL-10 vs. tissue MCP-1. Usually, TNF-α drives the production of IL-6 (De Cesaris et al. 1998) and VCAM-1 is elevated in response to inflammation (Lino et al. 2019), an occurrence that appears to be missing here. IL-6 is crucial in the switch from the initial inflammatory phase to the anti-inflammatory reparative phase. The lower levels of tissue IL-6 relative to comparators may therefore diminish the capability of the muscle to repair damage. As serum levels of early inflammatory markers increased, so did the associated tissue markers of damage and fibrosis, with positive correlations between both serum IL-6 and serum IL-1Ra vs. tissue collagen III, and serum IL-10 vs. tissue actin. Overall, these findings suggest a relatively insufficient anti-inflammatory response at tissue level, which may hamper tissue repair (Moon et al. 2023; Nakao and Libby 2023; Ollewagen et al. 2021). This hypothesised phenomenon of a gradual and cumulative reduction in endogenous counter systems against the backdrop of chronic stimulus, is not unique. A similar relative reduction in antioxidant defences in the face of chronically elevated oxidative stress is well known (Birben et al. 2012; Sharifi-Rad et al. 2020).

With regard to myocarditis, IL-18 has been implicated in both lupus and viral myocarditis (Fairweather et al. 2005; Du Toit et al. 2021). The correlation between TNF-α and IL-18 was observed in both serum and tissue, confirming perpetuation of auto-immune-associated systemic inflammation into the tissue. This is in line with a previous suggestion that this self-reinforcing pro-inflammatory loop is responsible for tissue damage in heart failure (Van Linthout and Tschöpe 2017). Similarly, an additional correlation in tissue between IL-1β and TNF-α (Rzeszotarska et al. 2022) suggests a persistent inflammatory environment in the tissue which aligns with active SLE.

In contrast to the serum vs. tissue picture, no significant correlations were observed between tissue IL-6 and other tissue pro- and anti-inflammatory markers, further leading us to hypothesize that its presence is limited relative to other tissue markers. These low IL-6 levels in tissue, and subsequent low IL-10 levels, suggest a relative scarcity of IL-6 at tissue level, which may result in an insufficient anti-inflammatory activation (Krishnamurthy et al. 2009). An ancestral link to decreased soluble IL-6 receptor (sIL-6R) has been reported for African populations (Reich et al. 2007), which is relevant to our study population. This receptor is known to facilitate IL-6 signalling in cells lacking membrane-bound IL-6 receptor via interaction with GP130, and to amplify signal when interacting with membrane-bound IL-6 (Chalaris et al. 2011) on cells expressing it (such as cardiac cells). Furthermore, plasma sIL-6R levels also correlate with IL-6 production capacity, and if this is reduced, the production of IL-6 is also affected (Chalaris et al. 2011; Evans and Abdou 1994). Thus, a relatively lower tissue IL-6 presence, combined with a potential ancestral lack of sIL-6R-amplification of IL-6 signalling, may provide a plausible explanation for the more aggressive form of LM that has been observed among patients of African ancestry. This notion is further supported by the preclinical literature. For example, IL-6 deficiency in rodents was reported to result in cardiac dysfunction by altering cell profiles (increasing fibroblasts and decreasing other cardiac cell populations), increasing the accumulation of interstitial collagen, altering cell-cell interactions, and decreasing the density of myocardial vasculature (Banerjee et al. 2009). Also, in the context of viral myocarditis, acute, but not long-term, IL-6 signalling was shown to be protective against acute damage (Fontes et al. 2015), illustrating the importance of maintaining optimal levels of IL-6.

Additionally, positive correlations exist in the tissue between both TNF-α and IL-1β vs. BMP-7, a negative regulator of pro-inflammatory cytokines (Narasimhulu and Singla 2020). BMP-7 may be an alternative pathway of activating the anti-inflammatory system. Based on literature, BMP-7 is expected to counter pro-inflammatory cytokines as well as TGF-β (for an anti-fibrotic effect) (Ollewagen et al. 2022; Singla et al. 2016; Wu and Hatzopoulos 2019). Current data illustrating BMP-7 to correlate with its triggers (TNF-α, IL-1β, TGF-β), but also with collagen I expression (i.e. the outcome it is aimed at preventing) suggest this response to be normally activated, but not sufficient to initiate a counter response. We have previously presented grounds for a cumulative gradual BMP-7 depletion as major role player in lupus nephritis (Smith et al. 2023). A longitudinal study may provide better insight and help elucidate the role of BMP-7 in ongoing myocardial inflammation.

Contextualising signalling data to clinical assessments, the negative correlations between tissue IL-6 and CMR characteristics of myocardial inflammation and necrosis/fibrosis (including T1, T2) appeared contradictory to its known role in inflammation (higher T1 and T2 is indicative of active inflammation and necrosis), which further supports our hypothesis of low IL-6 at a tissue level. In the clinical setting, the T1 and T2 scores are derived from observations of edema on imaging, which is arguably more accurately reflecting *early* phase inflammation (vasodilation and fluid accumulation in tissue, i.e. edema), which precedes immune cell infiltration, IL-6 secretion and IL-6-dependent signalling outcomes. However, in parallel, significant correlations between SOC markers and other tissue inflammatory and pro-fibrotic cytokine levels (e.g. aRo vs. tissue IL-1β and TGF-β and aLa vs. tissue TGF-β and BMP-7) supports the proposed interplay between myocardial inflammation and fibrosis observed in the tissue. Correlations between anti-Ro (aRo) antibodies and IL-1β have been well described across a range of connective tissue diseases, particularly in association with cardiac conduction abnormalities (Pisoni et al. 2015). The positive correlation observed between aRo antibodies and tissue TGF-β – a key marker of fibrosis - supports the established association of aRo antibodies with fetal heart block, as well as conduction abnormalities observed in adult patients with anti-Ro–positivity (Bourré-Tessier et al. 2011; Brucato et al. 2001).

Current data demonstrated the presence of LGE on CMR in seven out of twelve patients in the SLE-LM (58.3%) and one out of twelve in the SLE group (8%). In the SLE-LM group, higher tissue VCAM-1 levels and somewhat higher tissue IL-6 levels were associated with the presence of LGE. However, more research is required to fully elucidate the value of LGE as indicator of tissue fibrosis in LM, as data on this marker is ambiguous. Previous research found LGE (indicating cell necrosis and/or fibrosis) not to be a prominent feature of acute LM when compared to that of viral myocarditis. Yet LGE is frequently observed in SLE patients with no prior history of LM (Luo et al. 2023; Du Toit et al. 2021). Also, LGE has been showed to decrease in some patients with acute myocarditis, not translating to persistent fibrosis on EMB (Aquaro et al. 2019).

As is often the case for rare diseases such as LM as well as the difficulty in obtaining cardiac tissue samples, cohort numbers were relatively small. However, given the alignment of generated data with relevant literature, in our opinion, this was not a limitation in the current study. The non-SLE control group had a slightly higher proportion of males, white and older participants. Males exhibit greater pro-inflammatory immune responses (while females have generally heightened adaptive immune activity, making them more susceptible to autoimmune disease) (Bose and Jefferies 2022; Schwartzman-Morris and Putterman 2012), which may have decreased effect sizes demonstrated between this group and the SLE groups. Additionally, older populations are more likely to have higher cytokine levels (Shortanbayev et al. 2014) and an African study demonstrated that white adults presented with higher cytokine levels than matched black adults (Crouch et al. 2020). However, taken together, the inflammatory profile of this group was most limited, and therefore is unlikely to have influenced interpretations made. We acknowledge that current data was generated on small biopsies from one specific area of the heart and thus should not be considered definitive proof of events across the heart. Given the invasive nature of biopsy sampling in living patients, it would be difficult to improve on this approach. We suggest that postmortem samples could be analysed to inform on the variability of tissue cytokine presence and signalling in different areas of the heart. In terms of serum cytokine levels, we also acknowledge that levels of these analytes may be influenced by factors (such as sampling time, treatment exposure, or comorbid conditions) that cannot be well-controlled in a clinical setting, and that no reference ranges exist for most of these to aid interpretation. However, as stated, our results correlate well with previous reports on similar cohorts, which supports the validity of the data and our hypotheses. We further recommend that follow-on studies to elucidate cytokine signalling patterns specifically, include assessment of cytokine localization to cellular structures, which were not possible in the current study due to the small sample size available and the relatively low magnification used.

## Conclusions

From the data presented, we hypothesise that a disconnect between systemic inflammatory signalling and myocardial tissue responses may exist in SLE. Such disparities between systemic markers and tissue pathology are consistent with observations in SLE, where clinical findings, serological markers and organ pathology are often discordant. This parallels findings in both lupus nephritis as well as subclinical LM. Novel data contributes to our hypothesis that limited tissue IL-6 expression (relative to other cytokines) and a resultant limited endogenous anti-inflammatory response, may underpin LM, at least in an African cohort.

This prioritises the identification and monitoring of cytokine pathways that mediate injury within the *tissue* of LM patients. Understanding these pathways is necessary to identify potential therapeutic targets that may not be identified through SOC and serum assessments alone. A longitudinal study design may further help elucidate the cumulative cytokine maladaptation in the context in LM.

## Supplementary Information

Supplementary Material 1.

## Data Availability

The datasets used and/or analysed during the current study are available from the corresponding author on reasonable request.

## Abbreviations

ACR: American College of Rheumatology
aLa: Anti-La
aRo: Anti-Ro
BMP: Bone morphogenetic protein
CK: Creatine kinase
CMR: Cardiac magnetic resonance imaging
CS: Corticosteroid
dsDNA: Double stranded deoxyribonucleic acid
DPX: Distyrene, Plasticizer, and Xylene
eGFR: Estimated glomerular filtration rate
ELISA: Enzyme-linked immunosorbent assay
EMB: Endomyocardial biopsy
ESR: Erythrocyte sedimentation rate
EULAR: European League Against Rheumatism
H&E: Haematoxylin and Eosin
IL: Interleukin
IQR: Interquartile range
LGE: Late gadolinium enhancement
LM: Lupus myocarditis
MCP: Macrophage chemoattractant protein
OCT: Optimal cutting temperature
PBS: Phosphate buffer saline
PBS-T: Phosphate buffer saline with tween
PBS-X: Phosphate buffer saline with triton-X
s: Serum
SD: Standard deviation
SLE: Systemic lupus erythematosus
SLEDAI: Systemic lupus erythematosus disease activity index
SLE-M: Systemic lupus erythematosus with clinically evident lupus myocarditis
SOC: Standard of care
SST: Serum separating tubes
STIR: Short tau inversion recovery
t: tissue
TGF: Transforming growth factor
TNF: α-tumor necrosis factor-α
U-PCR: Urine protein creatinine ratio
VCAM: Vascular cell adhesion molecule
