# Supplementary material for: Serum vs. tissue cytokine dysregulation in SLE-associated myocarditis: a hypothesis from an African cohort perspective
Source: Mol Med. 2026 May 21;32:127. doi: 10.1186/s10020-026-01504-6 (PMC13474735; doi:10.1186/s10020-026-01504-6)
Supplement: Supplementary file 1 [file 10020_2026_1504_MOESM1_ESM.docx]

**Supplementary data**

Supplementary Figure 1: Representative images of primary antibody controls (primary antibody only), secondary antibody controls (secondary antibody only) and a tissue control (no antibodies) to determine autofluorescence and set microscope exposure levels. Only fully validated antibodies were used therefore no positive control staining was performed.

Supplementary Table 1: Additional organ manifestations according to SLEDAI-2K criteria. Corresponds with data presented in table 1

| **Organ manifestation** | **SLE** | **SLE-M** |
| --- | --- | --- |
|  | n=13 | n=14 |
| Seizure; n (%) | 1/13 (8) | 0/14 (0) |
| Psychosis; n (%) | 0/13 (0) | 0/14 (0) |
| Organic brain syndrome; n (%) | 1/13 (8) | 1/14 (7) |
| Visual disturbance; n (%) | 0/13 (0) | 0/14 (0) |
| Cranial nerve disorder; n (%) | 0/13 (0) | 0/14 (0) |
| Lupus headache; n (%) | 0/13 (0) | 0/14 (0) |
| CVA; n (%) |  |  |
| Vasculitis; n (%) | 1/13 (8) | 1/14 (7) |
| Arthritis; n (%) | 2/13 (15) | 7/14 (50) |
| Myositis; n (%) | 1/13 (8) | 1/14 (7) |
| Granular urinary casts; n (%) | 0/13 (0) | 0/14 (0) |
| Hematuria; n (%) | 0/13 (0) | 1/14 (7) |
| Pyuria; n (%) | 0/13 (0) | 0/14 (0) |
| Proteinuria; n (%) | 6/13 (46) | 8/14 (57) |
| Rash; n (%) | 5/13 (38) | 8/14 (57) |
| Alopecia; n (%) | 5/13 (38) | 7/14 (50) |
| Mucosal ulcers; n (%) | 2/13 (15) | 2/14 (14) |
| Pericarditis; n (%) | 1/13 (8) | 2/14 (14) |
| Pleuritis; n (%) | 1/13 (8) | 4/14 (29) |
| Increased ads DNA; n (%) | 10/13 (77) | 12/14 (86) |
| Low complement; n (%) | 5/10 (50) | 11/14 (79) |
| Fever; n (%) | 1/11 (9) | 1/14 (7) |
| Platelets <100 000; n (%) | 0/13 (0) | 4/14 (29) |
| WCC < 3; n (%) | 4/13 (31) | 1/14 (7) |
| Lupus lung; n (%) | 0/13 (0) | 0/14 (0) |
| Lupus nephritis; n (%) | 4/13 (31) | 5/14 (36) |
| NYHA dyspnea grade 1-4; n (%) | 3/13 (23) | 10/14 (71) |
| Orthopnea; n (%) | 0/12 (0) | 5/14 (36) |
| Palpitations; n (%) | 1/12 (8) | 1/14 (7) |

Supplementary Figure 2: Comparison of treatment with high prednisone vs low/no prednisone on serum cytokine parameters in both systemic lupus erythematosus (SLE) cohorts combined. High pred – Solumedrol 3 day pulse/Prednisone >1/2 to 1 mg/kg/day; Low/no pred – Prednisone <1/2 mg/kg/day. Data presented as median±IQR. No significant differences noted.

Supplementary Figure 3: Correlations between serum parameters in all groups. Graphs demonstrate significant results/trends and present p-values and r-values. Corresponds with data presented in table 2.

Supplementary Figure 4: Correlations between serum (x-axis) and tissue (y-axis) parameters in systemic lupus erythematosus (SLE) myocarditis patients. Graphs demonstrate significant results/trends and present p-values and r-values. Corresponds with data presented in Table 3.

Supplementary Figure 5: Correlations between different tissue parameters in systemic lupus erythematosus (SLE) myocarditis patients. Graphs demonstrate significant results/trends and present p-values and r-values. Corresponds to data presented in table 4.

Supplementary Figure 6: Correlations between tissue parameters and standard clinical markers in systemic lupus erythematosus (SLE) myocarditis patients. Graphs present significant differences/trends and indicate p-values and r-values. Corresponds with data presented in table 5.

Supplementary Figure 7: Correlations (A-D) between tissue parameters and CMR data in systemic lupus erythematosus (SLE) myocarditis patients (Graphs present significant differences/trends and indicate p-values and r-values). E) Graph representing the VCAM-1 integrated density difference between presence (1) and absence (2) of LGE. *p<0.05. Data corresponds with Table 6.
